# Uptake of pH-Sensitive Gold Nanoparticles in Strong Polyelectrolyte Brushes

**DOI:** 10.3390/polym8040134

**Published:** 2016-04-08

**Authors:** Dikran Kesal, Stephanie Christau, Patrick Krause, Tim Möller, Regine von Klitzing

**Affiliations:** Stranski-Laboratorium für Phyisikalische Chemie, Technische Universität Berlin, Straße des 17. Juni 124, 10623 Berlin, Germany; dikran.kesal@campus.tu-berlin.de (D.K.); stepchri@umich.edu (S.C.); p.krause_22.09.1991@mailbox.tu-berlin.de (P.K.); timmeymoeller@mail.tu-berlin.de (T.M.)

**Keywords:** polymer brushes, gold nanoparticles, nanocomposite, polyelectrolyte, surface plasmon resonance, electrostatic interaction, smart surfaces, surface-initiated atom transfer radical polymerization (Si-ATRP)

## Abstract

The impact of electrostatic attraction on the uptake of gold nanoparticles (AuNPs) into positively charged strong poly-[2-(Methacryloyloxy) ethyl] trimethylammonium chloride (PMETAC) polyelectrolyte brushes was investigated. In this work, PMETAC brushes were synthesized via surface-initiated atom transfer radical polymerization (Si-ATRP). PMETAC/AuNP composite materials were prepared by incubation of the polymer brush coated samples into 3-mercaptopropionic acid-capped AuNP (5 nm in diameter) suspension. The electrostatic interactions were tuned by changing the surface charge of the AuNPs through variations in pH value, while the charge of the PMETAC brush was not affected. Atomic-force microscopy (AFM), ellipsometry, UV/Vis spectroscopy, gravimetric analysis and transmission electron microscopy (TEM) were employed to study the loading and penetration into the polymer brush. The results show that the number density of attached AuNPs depends on the pH value and increases with increasing pH value. There is also strong evidence that the particle assembly is dependent on the pH value of the AuNP suspension. Incubation of PMETAC brushes in AuNP suspension at pH 4 led to the formation of a surface layer on top of the brush (2D assembly) due to sterical hindrance of the clustered AuNPs, while incubation in AuNP suspension at pH 8 led to deeper particle penetration into the brush (3D assembly). The straightforward control of particle uptake and assembly by tuning the charge density of the nanoparticle surface is a valuable tool for the development of materials for colorimetric sensor applications.

## 1. Introduction

The modification of surfaces with thin films is widely used to tailor surface characteristics, which define the physical and chemical properties and can provide smart surfaces with desired properties. Polymer brushes represent a class of thin films where the polymer chains are chemically end-grafted to the substrate. The chain functionality can be tailored by chemical composition and allows the brushes responding to outer stimuli like temperature [1,2,3,4], pH [5,6,7,8,9], ionic strength [10,11,12,13,14] or solvent [15,16,17,18]. Due to their responsive nature, brushes are highly applicable for their use as “smart” surfaces. They can be found in many applications for coatings, for instance as antifouling surfaces in biotechnological applications [19,20,21,22]. They are also potential candidates for drug delivery systems [23,24,25,26,27].

Besides the responsiveness, polymer brushes have a positive influence on the steric stabilization of colloids, which make them suitable candidates as a matrix for the incorporation of inorganic nanoparticles like gold nanoparticles (AuNPs). AuNPs induce optical properties due to their surface plasmon resonance (SPR), which results in smart nanocomposite materials with tunable optical properties for application as sensors [28]. The SPR occurs from the collective oscillation of the conduction electrons of AuNPs due to the interaction with the photons of the incident light, which results in the typical color of nanoparticle suspensions [29]. The SPR absorption band depends on a variety of parameters such as size, shape, surface coating or the interparticle distance, and it can be detected through spectroscopical measurements, like UV/Vis spectroscopy [30]. Decreasing the interparticle distance leads to a shift of the plasmon peak to larger wavelength (red-shift), while an increase leads to the opposite shift to lower wavelengths (blue-shift) [31]. Therefore, the local particle number density of AuNPs has a significant impact on the interparticle distance within polymer brushes. Christau *et al.* [32] studied the effect of brush thickness on AuNP uptake. They found an increased particle uptake with increasing polymer molecular weight due to increased surface roughness. Furthermore, they reported that the type of assembly was not affected by the brush thickness. In particular, the majority of AuNPs were attached on top of the brush and only a low amount of particles was found to penetrate the brush. Further studies by Christau *et al.* [33] elaborated that changing the grafting density has a dramatic impact on the assembly of AuNPs within polymer brushes, with maximum particle uptake for intermediate grafting densities. The particle distribution was rather homogeneous within the whole brush (3D assembly). A more likely 2D assembly was found for high grafting densities, where the high osmotic pressure in the brush and steric hindrance limited the particle attachment and penetration. Recent observations have shown that the type of particle assembly (2D or 3D) has a strong effect on collective properties. For example, pronounced IR transparency and electrical insulation were found for AuNPs assembled as a layer (2D), and a decrease in electrical resistance was found for AuNPs assembled in 3D [34,35].

In order to control collective properties, both electronical and optical ones, it is important to understand how to control the distribution of the AuNPs. One important parameter is the charge of the AuNP, which is assumed to drive the adsorption and penetration of AuNP into the brush [32,36].

In the present paper, we addressed this aspect by studying the effect of charge of AuNPs, on their adsorption into a strong positively charged brush. The 5 nm AuNPs were coated with 3-mercaptopropionic acid (3-MPA) instead of citrate, since thiol-gold bonds are more stable than the physiosorbed citrate ions [37]. In addition, 3-MPA carries a carboxylic acid group that can be protonated or deprotonated, which allows for charging or uncharging the surface of the AuNPs by changing the pH. For separating the effect of AuNP charge from brush properties, it is necessary that the brush charge remains constant. Therefore, poly-[2-(Methacryloyloxy) ethyl] trimethylammonium chloride (PMETAC), a strong positively charged polyelectrolyte brush, was used. A combination of ellipsometry, atomic-force microscopy, UV/Vis spectroscopy, gravimetric analysis, and transmission electron microscopy allowed for obtaining a complete picture of particle loading and penetration of AuNPs inside the PMETAC brushes.

## 2. Experimental Section

### 2.1. Materials

The chemicals that are used for the synthesis were received from Sigma-Aldrich (St. Louis, MO, USA) and were used without further purification.

### 2.2. Synthesis

The polymer brushes have been synthesized via the grafting from method through the surface-initiated atom transfer radical polymerization (Si-ATRP). Si-ATRP is a controlled radical polymerization technique which provides a number of advantages over traditional free radical techniques like the control over the brush thickness and the low polydispersity [38,39]. In order to yield polymer brushes, two synthesis steps are needed. As a first step, an initiator-coated self-assembled monolayer (SAM) has to be generated, and as a second step, the surface-initiated polymerization is carried out.

For both generating of the SAM and the synthesis of the brush, a specific reactor was used, which is described elsewhere [32]. The advantage of this reactor is that various samples can be prepared at the same time in order to achieve the same conditions for all samples.

#### 2.2.1. Preparation of Buffer Solutions with Different pH

Buffer solutions were used to obtain the desired pH because they maintain a relatively constant pH over a long period. Low ionic strength (∼0.05 M) buffer solutions were selected to guarantee that no salt effects disturb the brush/AuNP composites in aqueous solution. Here, three different buffer systems (formic acid/KOH, succinic acid/KOH and trishydroxymethylaminomethane (TRIS)/HCl) were used. For pH 4, 15.03 mL of formic acid (0.1 M) were mixed with 9.89 mL of KOH (0.1 M) and were filled up to 100 mL with ultrapure water (Milli-Q water). For pH 6, 20.07 of succinic acid (0.02 M) and 14 mL of KOH (0.05 M) were mixed together and were filled up to 100 mL with Milli-Q water. For pH 8, 15.84 mL of TRIS (0.1 M) were mixed with 10 mL of HCl (0.1 M) and were filled up to 100 mL with Milli-Q water.

#### 2.2.2. Synthesis of the Initiator

As the initiator, 2-bromo-2-methyl-*N*-(3-(triethoxysilyl)propyl)propan- amide (BTPAm) was used. BTPAm was prepared according to literature procedure [40] by a coupling reaction between aminopropyltriethoxysilane (APS) with 2-bromoisobutyrylbromide (BIBB) through an amidiziation reaction.

#### 2.2.3. Immobilization of the Initiator BTPAm onto the Surface by Building a SAM

The substrates were etched for 30 min using piranha solution (H_2_SO_4_/H_2_O_2_ 1:1 *v/v*%). This procedure generates hydroxyl groups on the surface to achieve a chemical bounding between surface and the initiator. After etching the samples, they were rinsed with Milli-Q water and the freshly cleaned substrates were placed into the reactor containing 10 mM solution of BTPAm in anhydrous toluene. The reactor was sealed and the reaction was carried out for 24 h at room temperature. After the reaction, the initiator-coated samples were rinsed with toluene, sonicated for 20 min in ethanol and then dried under a stream of N_2_.

#### 2.2.4. Synthesis of Poly-2-(methacryloyloxy)ethyltrimethylammonium Chloride (PMETAC) by Si-ATRP

The polymerization through Si-ATRP was followed literature procedure [41], but the literature recipe was modified by changing the CuCl/CuCl_2_ and changing the concentration of the monomer 2-(methacryloyloxy)ethyltrimethylammonium chloride (METAC) in order to decrease the polymerization time. In a typical reaction protocol, METAC with various amounts (20 mL, 85 mmol; 35 mL, 150 mmol; 47 mL, 200 mmol) was mixed with 2,2’-bipyridyl (844 mg, 5.4 mmol) in 20 mL of Milli-Q water/MeOH (1:4 *v/v*) in the reactor. The mixture was flushed with N_2_ for 30 min. Then, CuCl and CuCl_2_ of different amounts (ratio of 10:1: 217.8 mg, 2.2 mmol and 30 mg, 0.22 mmol or 15:1: 326.7 mg, 3.3 mmol and 30 mg) was added quickly to the mixture. The mixture was further stirred and degassed for another 30 min. The initiator-coated samples were placed in the reactor in nitrogen atmosphere. The polymerization was carried out for a given polymerization time at room temperature. By exposing the samples to air, the polymerization can be stopped after that time. The samples were sonicated in water for 10 min, then in MeOH for another 10 min and dried with N_2_.

#### 2.2.5. Synthesis of AuNPs

3-Mercaptopropionic acid-capped (3-MPA) AuNPs were synthesized by modifying a multi-step procedure similar to the procedure described in [42]. As a first step, citrate stabilized AuNPs were prepared. Here, a solution of gold(III) chloride hydrate (HAuCl_4_ · *x*H_2_O) (0.25 mL, 0.1 M) was added to 99.5 mL of Milli-Q water and was stirred with a solution of trisodium citrate dihydrate (C_6_H_5_Na_3_O_7_·2H_2_O) (0.25 mL, 0.1 M) to the previous solution. While the mixture was stirred, a solution of sodium borohydride (NaBH_4_) (2 mL, 0.1 M) was added into this at once. The solution turned red immediately. The AuNP suspension was stirred for another 2 h. In order to gain 3-MPA-capped AuNPs, the citrate molecules on the AuNP surface were displaced by thioctic acid. For this purpose, thioctic acid (6 mg, 0.029 mmol) was dissolved in 1 mL ethanol and added to the previously synthesized citrate reduced AuNPs while the suspension was stirred. The suspension turned their color from red to purple. NaOH solution was added dropwise to the purple suspension until the suspension color changed to red again. The mixture was stirred overnight so that the reaction could reach equilibrium. The last step was the functionalization of the AuNPs with 3-MPA by adding 2.18 μL (0.025 mmol) to the suspension and letting it stir overnight. In order to get rid of the excess ligand, the AuNPs were precipitated by adding ethanol to the AuNP suspension. The suspension was centrifuged at 7000 rpm for 20 min. The supernatant was discarded while the pellet was resuspended in Milli-Q water with a pH of 13 prior to use (Supplementary Materials, Figure S1). 3-MPA AuNP suspensions have a pK_a_ of 4.3 (see ref [43]) (Supplementary Materials, Figure S2). The diameter of the particles were found to be 4.8 ± 1.1 nm, as determined by transmission electron microscopy (TEM) and using ImageJ (National Institutes of Health, Rockville, MD, USA) for the size determination (Supplementary Materials, Figure S4).

#### 2.2.6. Preparation of PMETAC/AuNP Brush Composites

Brush/AuNP composites can be achieved by incubation of the PMETAC brushes into the AuNP suspension. The samples were incubated for 24 h. After incubation, the samples were taken out, sonicated in Milli-Q water for 5 min and dried under a N_2_ stream. The PMETAC brushes were incubated at pH 4, at pH 6 and at pH 8 by mixing AuNP stock suspension with buffer solution at a 1:9 AuNP/buffer ratio.

### 2.3. Instruments and Measurement Procedure

#### 2.3.1. Ellipsometry Measurements

The ellipsometric measurements were carried out with a polarizer-compensator sample analyzer (PCSA) ellipsometer (Optrel GbR, Sinzing, Germany) at a wavelength of 623.8 nm in null ellipsometry mode. The measurements were carried out at an angle of incidence of 70° under ambient conditions (relative humidity (rh) ≈ 30%) and at an angle of incidence of 60° for measurements in water. The data were fitted with Elli v3.1 (Optrel, Sinzing, Germany) based on the following two-layer-model shown in Table 1.

#### 2.3.2. Atomic-Force Microscopy (AFM) Measurements

Scanning measurements were carried out with a Cypher AFM (Asylum Research, Santa Barbara, CA, USA) at a scan rate of 1 Hz. For measurements in air, silicon cantilevers with a reflective coating of aluminum (AC160TS, Olympus, Tokyo, Japan) were used. The cantilever had a length of 160 μm, a resonant frequency of 300 kHz and a force constant of 42 N/m. Measurements in water were performed with Cr/Au-coated silicon cantilevers (OMCL-TR series, Olympus, Tokyo, Japan) with a triangular shape, a resonant frequency of 11 kHz and a spring constant of 0.02 N/m. All measurements were done at room temperature. The data were analyzed with IgorPro (Wavemetrics, Inc., Portland, OR, USA). The roughness was determined by the average of the root mean square roughness (rms) of 3 areas of (20 × 20) μm^2^. The rms was calculated using the formula:

(1) $$\sigma =\sqrt{\left(\frac{1}{N}\sum y_{i}^{2}\right)},$$

where *N* is the number of data points and *y_i_* the respective height of each data point. All images were planar fitted and flatted to correct any tilting of the samples.

The full-indentation method were used to measure the thickness in water, which has been already introduced by Üzüm *et al.* [44] for polyelectrolyte multilayers and were applied on polymer brushes doped with AuNPs by Yenice *et al.* [45]. An AFM force measurement setup was used for indentation in *z*-direction. The experiments were carried out in water at room temperature on an MFP-3D AFM (Asylum Research, Santa Barbara, CA, USA). Cr-Au coated silicon cantilevers (HQ:CSC38/CR-Au, MikroMasch, Sofia, Bulgaria) with a spring constant of 0.05 N/m were used. Each sample measurement consists of 12 individual force curves taken at different lateral positions on the sample. Details are described in ref [44,45].

#### 2.3.3. TEM Measurements

TEM measurements were performed on a FEI Tecnai G^2^ 20 S-TWIN (FEI, Hillsboro, OR, USA). Average gold core diameter (*D*), size distributions and standard deviations were calculated for each AuNP by averaging 200 particles from the TEM images using ImageJ.

#### 2.3.4. Gravimetric Analysis

Gravimetrical measurements were performed to determine the amount of AuNPs in the stock suspension. To do this, 1 mL of the AuNP stock suspension was poured into a porcelain crucible. The vessel was placed in a muffle furnace and a defined temperature program was run to guarantee that everything was decomposed except the gold. The amount of gold of the used AuNP stock suspension volume can be determined by weighing the vessel before pouring AuNP stock suspension and after the decomposition of the AuNP stock suspension which has been done three times (detailed calculations will be described in Section 3.2 Effect of pH on AuNP Dispersion and in Supplementary Materials).

#### 2.3.5. UV-Vis Spectroscopy

Spectroscopic measurements were performed in a wavelength range of 400–800 nm at room temperature in aqueous solution using a UV/Vis spectrophotometer (PerkinElmer Inc., Waltham, MA, USA). In order to record UV/Vis spectra and measure the transmittance of PMETAC/AuNP composites, the substrate from silicon wafer to fused silica wafer (Microchemicals GmbH, Ulm, Germany) was changed.

## 3. Results

### 3.1. PMETAC Brush

#### 3.1.1. Tuning of the Brush Thickness

**Figure 1:**
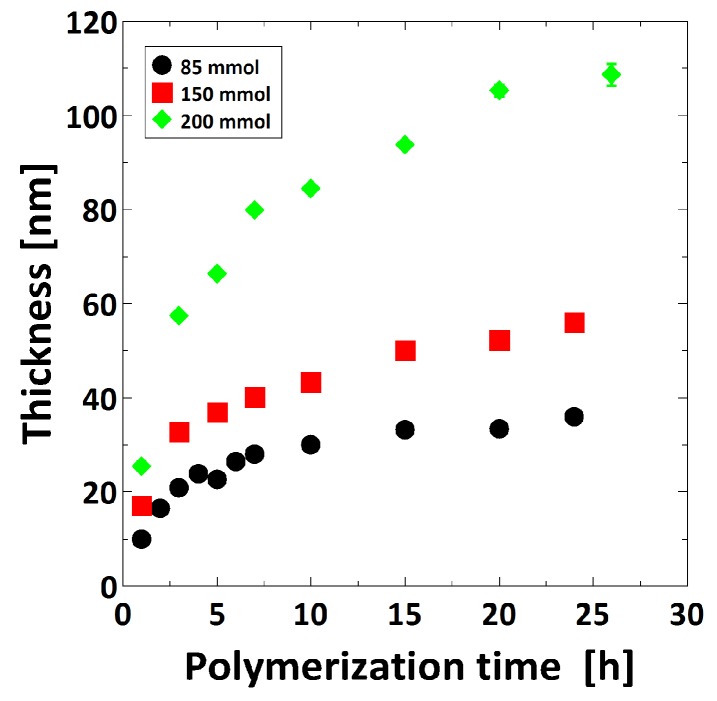
Dependence of poly-[2-(Methacryloyloxy) ethyl] trimethylammonium chloride (PMETAC) brush thickness with polymerization time and monomer concentration measured at ambient conditions by ellipsometry.

The brush thickness was controlled by tuning polymerization time, ratio of CuCl/CuCl_2_ and monomer concentration. The thickness was measured at ambient conditions by ellipsometry. Both for the CuCl/CuCl_2_ ratio of 10:1 and 15:1 the brush thickness reaches a plateau after several hours (see Supplementary Materials, Figure S3). Using a ratio of 15:1 (CuCl/CuCl_2_) slightly thicker polymer brushes could be prepared compared to the ratio of 10:1, using the same polymerization time. The monomer concentration was adjusted to three values at a constant CuCl/CuCl_2_ ratio of 15:1. The brush thickness increases with increasing polymerization time as well as the monomer concentration (Figure 1).

**Figure 2:**
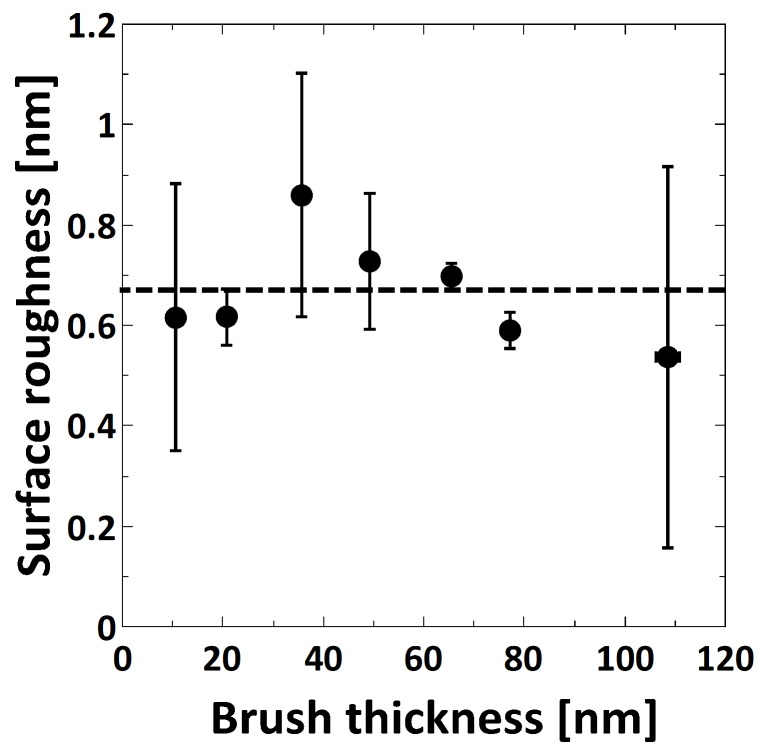
Surface roughness at ambient conditions (determined from atomic-force microscopy (AFM) micrographs) as a function of ambient brush thickness (as measured by ellipsometry).

The surface roughness was determined using AFM topographical images measured at ambient conditions. Within the measured brush thickness range, the surface roughness is constant with a value of 0.7 ± 0.1 nm (Figure 2).

#### 3.1.2. Neat PMETAC Brush in Ambient Conditions and Water

In the present study, PMETAC brushes with thickness around 30 nm (ambient conditions) were used for the attachment of the particles. The samples were obtained from a monomer concentration of 85 mmol and a Cu(I)/Cu(II) ratio of 15:1. The thickness measured under ambient condition for different neat PMETAC brushes (sample 1–4) are listed in Table 2. All samples are from the same batch. They were synthesized simultaneously in the reactor under exact the same conditions.

The samples 2–4 were used to prepare PMETAC/AuNP composite materials by incubating sample 2 into the AuNP suspension at pH 4, sample 3 into the AuNP suspension at pH 6, and sample 4 into the AuNP suspension at pH 8, while sample 1 was used as a reference for a neat brush. The brush thickness in Milli-Q water was also measured for sample 1, both by ellipsometry and by full-indentation AFM (Table 3).

**Figure 3:**
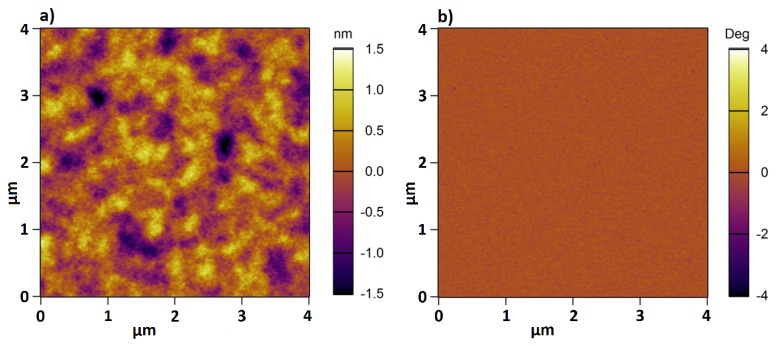
AFM height (**a**) and phase (**b**) image for sample 1 in Mili-Q water at room temperature.

As demonstrated in Table 3, the results obtained by the two independent methods are consistent within the experimental errors. This demonstrates that the full-indentation method can be also used to measure the thickness of polymer brushes. Beside thickness determination, AFM can be used in scanning mode to gain knowledge about the surface topography and material properties. Figure 3 shows the height (a) and phase (b) image for sample 1 recorded in Milli-Q water at room temperature. The height image reveals a homogeneous surface topology, while the phase image demonstrates a homogeneous composition for the PMETAC brush.

### 3.2. Effect of pH on AuNP Dispersion

#### 3.2.1. Particle Shape and Size at Different pH

**Figure 4:**
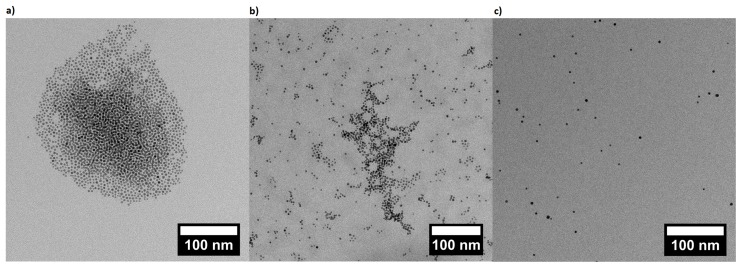
Transmission electron microscopy (TEM) measurements of gold nanoparticles (AuNPs) dispersed (**a**) at pH 4; (**b**) at pH 6; (**c**) at pH 8.

AuNP suspensions at different pH were prepared (AuNP at pH 4, at pH 6 and at pH 8) by mixing AuNP stock suspension with the buffer solution at the ratio of 1:9. AuNP suspensions at pH 4, at pH 6 and at pH 8 were investigated by TEM measurements and are shown in Figure 4. AuNPs at pH 8 reveals a homogenous distribution of individual particles. By decreasing the pH to 6, it can be noticed that the clustering begins with building networks and finally, at pH 4, the particles form small clusters. However, AuNPs at pH 4 are still spotted as single particles.

#### 3.2.2. UV/Vis Spectroscopy Characterization

**Figure 5:**
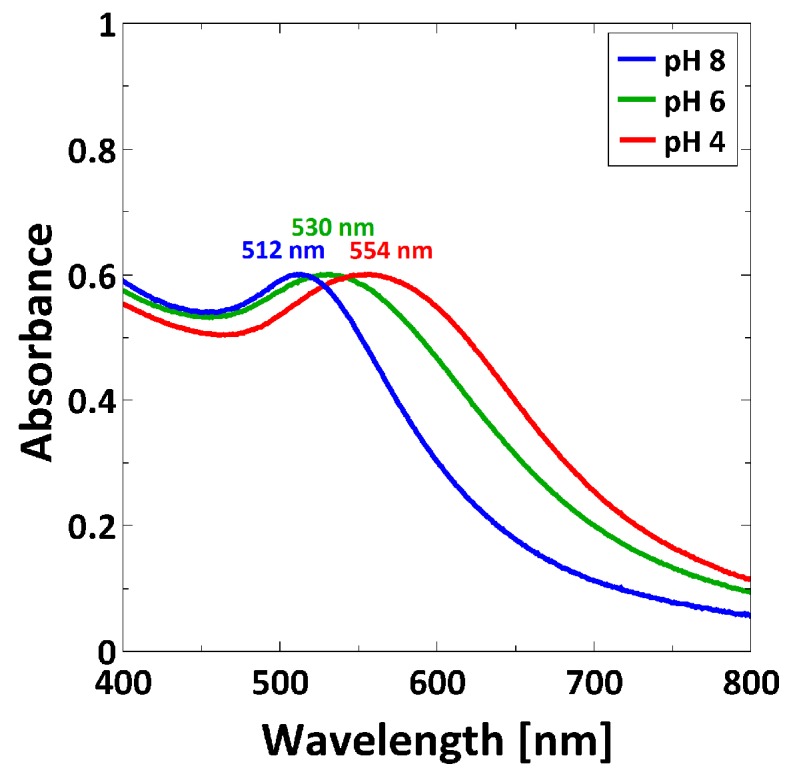
UV/Vis spectra recorded at room temperature for AuNP suspensions at pH 4, at pH 6 and at pH 8.

AuNPs have the ability to interact with light through a collective oscillation of the conduction electrons on the metal surface, known as surface plasmon resonance (SPR). This phenomenon can be detected by UV/Vis spectroscopy [46]. AuNP suspensions at pH 4, at pH 6 and at pH 8 were measured at room temperature (Figure 5). As shown in Figure 5, the surface plasmon peak shifts to longer wavelengths according to a decrease of pH.

#### 3.2.3. Gravimetric Analysis of AuNP Concentration

The several synthesis steps necessary to produce 3-MPA-capped AuNPs lead to some loss of product. This implies that the calculation of the AuNP concentration from the initial amount of HAuCl_4_ would be too inaccurate. Therefore, the gold amount has been determined three times by gravimetric analysis of the 3-MPA-capped AuNPs. The amount of gold (*m*_gold_) in 1 mL AuNP suspension is (1.6 ± 0.4) ×10^−3^ g. From this, the concentration (*c*) of the incubation suspension at different pH can be calculated by estimating the total amount of AuNPs (*n*_total_) in 1 mL of AuNP stock suspension (see Supplementary Materials for detailed calculations). The incubation suspensions were obtained by mixing 1 mL of AuNP stock suspension (*n*_total_ = 2.45 nmol) with 9 mL of buffer solution. Therefore, *c* of the incubation suspension is 0.245 nmol/mL. According to Lambert–Beer law (Equation (2)), the absorbance is proportional to the concentration:

(2) A=clϵ,

where *l* is the length of the cuvette the light passes through, *ϵ* is the extinction coefficient, *c* the concentration and *A* the absorbance. Therefore, *c* can be directly linked to the corresponding SPR absorbance peak measured by UV/Vis spectroscopy. Finally, the concentration within polymer brushes can be indirectly calculated by measuring the absorbance of the incubation medium before and after incubation. This procedure will be described more in detail in the next section (see Section 3.3.3).

### 3.3. Composite Material of PMETAC/AuNP

#### 3.3.1. Characterization of PMETAC/AuNP Composites by AFM

**Figure 6:**
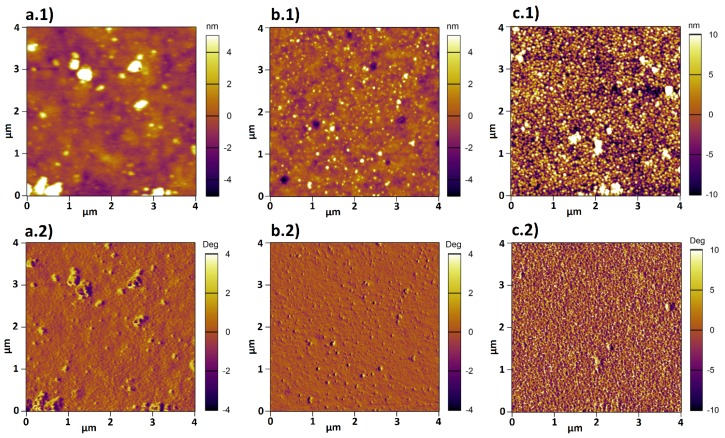
Height (**a.1**-**c.1**) and phase (**a.2**-**c.2**) images were recorded in water by AFM for composites (**a.1**/**a.2**) at pH 4, (**b.1**/**b.2**) at pH 6, (**c.1**/**c.2**) at pH 8.

AFM was used to investigate both morphology (by scanning) and thickness (by full-indentation) of the composite brushes. Surface topography and phase images of composites at different pH are shown in Figure 6.

Two trends were observed in the height images a.1-c.1 shown in Figure 6. First, the amount of deposited AuNPs on top of the brush increases with increasing pH. Second, the tendency to cluster decreases for increasing pH. Furthermore, the phase images reveal that two different materials are present, because the phase signal changes when the probe encounters regions with different composition.

After AuNP deposition, the thickness of the composites cannot be determined with monochromatic ellispometry, due to the dielectric function of the AuNPs as a new parameter. Therefore, the thickness was determined in water by the full-indentation method. The values for the composites incubated at pH 8, at pH 6 and at pH 4 are listed in Table 4. The thickness of the neat brush is also reported for comparison.

The swollen thickness of the samples increases after loading with AuNPs. Furthermore, the values increases with increasing pH of the AuNP suspension.

#### 3.3.2. UV/Vis Characterization

**Figure 7:**
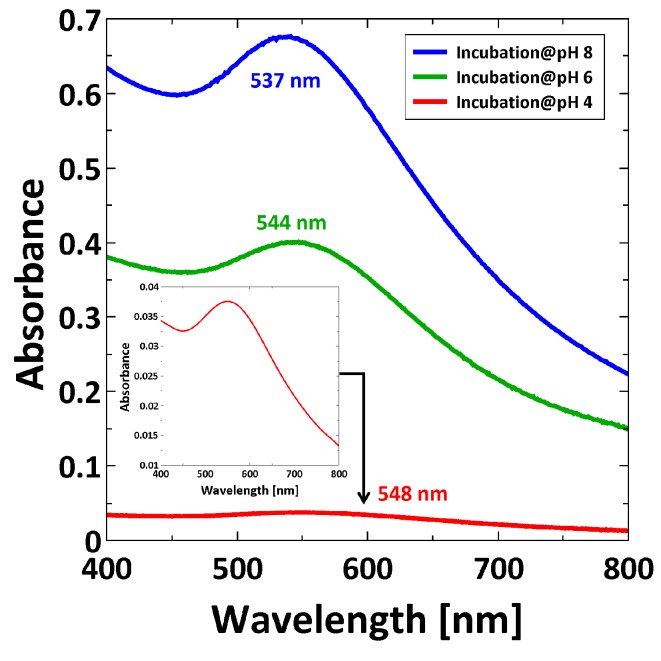
UV/Vis spectra recorded in water for PMETAC/AuNP composites incubated at pH 4, at pH 6, and at pH 8. Magnified representation of the SPR band for PMETAC/AuNP composite at pH 4 (inset).

After incubation in AuNP suspension, the PMETAC/AuNP composites were analyzed by UV/Vis spectroscopy. The plasmon band is analyzed as follows: the intensity is proportional to the amount of particle content, while the position of the plasmon peak gives information about SPR coupling [47,48]. Here, PMETAC brushes incubated at different pH were measured in water, and the corresponding absorbance peaks are reported in Figure 7.

Figure 7 reveals two trends. First, the intensity of the plasmon peak is decreasing for decreasing pH, which is a typical indication for lower particle uptake with decreasing pH. Second, the plasmon peak is shifted to a higher wavelength with decreasing pH, which indicates a decrease in the interparticle distance [49].

#### 3.3.3. The Amount of AuNPs within PMETAC Brushes

**Figure 8:**
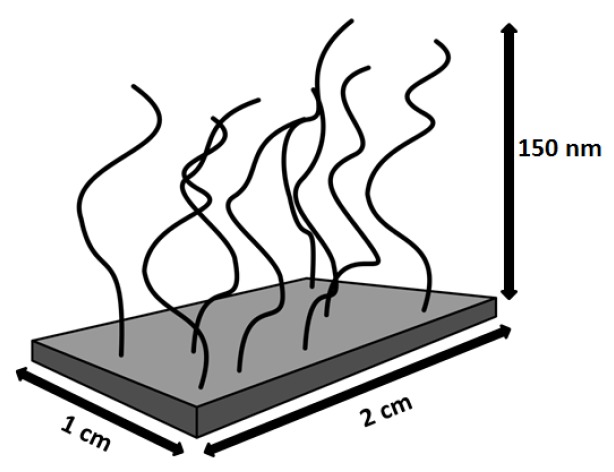
Determination of particle number densities using a box model.

The amount of AuNPs adsorbed in the PMETAC brushes was estimated from the difference of the AuNP concentration before and after brush incubation. In fact, from the measurement of the absorbance by UV/Vis spectroscopy of the AuNP suspensions before and after the incubation (see Supplementary Materials, Figure S5) a decrease of the absorbance after the incubation was found, which was explained by particle uptake. Since the amount of AuNP was measured by gravimetric analysis, *c* could be linked to the absorbance through Lambert–Beer’s law (Equation (2)). Therefore, the surface plasmon peak before and after incubation can be used to determine the particle number density (Equation (3)) within PMETAC, since *A* ∼ *c*. Using the calculated amount of AuNPs within the brush, it is possible to calculate the respective particle number densities. For this purpose, a box model (Figure 8) was used. The calculated particle number densities are listed in Table 5.

The difference between *c* after incubation and *c* before incubation is defined as Δ*c*, which can be calculated back to Δ*N_total_* with eq. S4. Δ*c* corresponds to the total amount of AuNPs within the PMETAC brush. Assuming that the brush is located on the whole glass slide (2 × 1 cm) and is 150 nm thick in swollen state (Figure 8), and assuming also a homogeneous AuNP distribution over the whole brush area, the volume of the incubated brush (*V_brush_*) can be calculated. Therefore, the particle number density (Equation (3)) is 1.1 × 10^−3^ particles/nm^3^ for the incubation of the brush at pH 4, 5.9 × 10^−3^ particles/nm^3^ at pH 6 and 8.3 × 10^−3^ particles/nm^3^ at pH 8. The particle number density of attached AuNPs increases with increasing pH of incubation suspension:

(3) $$Particle\;number\;density=\frac{\Delta N_{total}}{V_{brush}}.$$

## 4. Discussion

In the present study, the thickness of the PMETAC brushes was tuned by means of CuCl/CuCl_2_ ratio, polymerization time and monomer concentration. In particular, the brush thickness increases with increasing CuCl/CuCl_2_ ratio, polymerization time and monomer concentration, although changing the CuCl/CuCl_2_ ratio did not result in significant changes (see Supplementary Materials, Figure S3). Similar growth behavior was observed for other polymers, like PDMAEMA for similar changes of CuCl/CuCl_2_ and polymerization time [32] and poly(oligo(ethylene glycol)acrylamide) (PMEGAm) brushes for increasing monomer concentration [50]. The surface roughness of the prepared brushes was found to be independent of the brush thickness, with an average surface roughness of 0.7 ± 0.1 nm in dry ambient conditions (Figure 2).

PMETAC brushes with around 30 nm in ambient conditions (Table 2) were used to study the particle uptake by changing the pH of the incubation medium. The reasons for using thin polymer brushes were manifold. Christau *et al.* observed AuNP crowding on PDMAEMA brushes with increasing polymer brush thickness (beyond 40 nm thickness in ambient conditions) [32], which made it challenging to obtain single particles attached to the surface. In addition, recently published results have shown that there are instances that polymer brushes tend to degraft by exposing them to a good solvent. Those include brushes with a high degree of charges (e.g., polyelectrolyte brushes) or containing nonpolymeric materials (e.g., NPs) [51,52,53]. To overcome the swelling-induced degrafting by reducing the tension on the brush, the molecular weight of the brushes was decreased.

3-MPA-capped AuNPs were synthesized by a ligand exchange reaction of citrate-covered AuNPs. They are characterized by pH-sensitivity (pK_A_ of 3-MPA-capped AuNPs is 4.3 [43]). TEM images (Figure 4) reveal that decreasing the pH decreases the interparticle distance due to the protonation of the carboxylic acid group on the surface, and therefore the reduction of electrostatic repulsions. AuNPs are still sterically stabilized through the capping, and can form clusters via the formation of H-bond of the protonated carboxylic acid groups (Figure 4c). Given that AuNPs exhibit SPR, which is dependent e.g., on the interparticle distance [54] through SPR coupling, UV/Vis measurements were carried out (Figure 5). The position of the SPR peak of 3-MPA-capped AuNPs depends on the pH. The shift of the peak to a higher wavelength for decreasing pH was explained by the decrease in the interparticle distance. This is in agreement with previous results from Lim *et al.* [55], where decreasing of the interparticle distance leads to a red-shift of the SPR peak.

**Figure 9:**
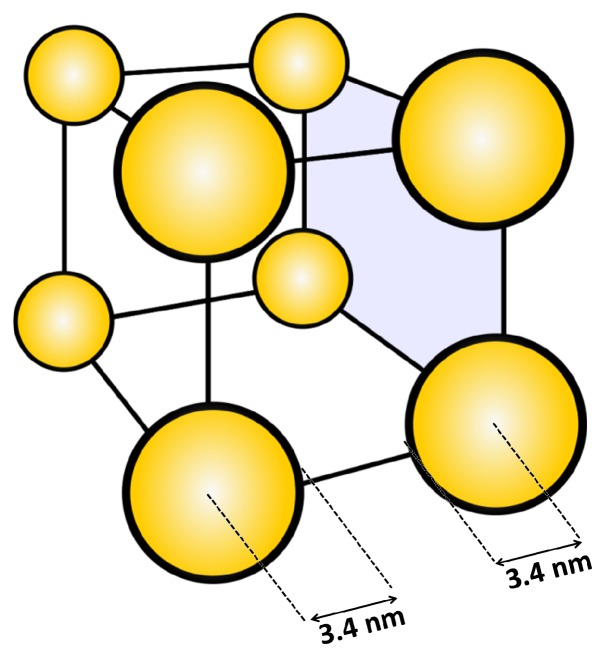
Distribution of the calculated number of particles for composites at pH 8 in a cube with a volume of (10 × 10 × 10) nm^3^.

Since strong polyelectrolytes like PMETAC are insensitive to pH and have permanent positive charges, they present a suitable matrix to study pH effects on the distribution of AuNPs with a pH-sensitive capping. For all studied pH, AuNP uptake could be observed, which causes an increase in brush thickness. The increase is explained by an increase in osmotic pressure due to an enhanced amount of counterions within the brush. The consequence is a stronger swelling in water (Table 4). Using gravimetric analysis and UV/Vis spectroscopy, the particle number density could be calculated. The results showed that the particle number density increases with increasing pH (Table 5), which is attributed to the more negatively charged AuNPs due to the deprotonation of the carboxylic acid groups of the 3-MPA capping. At pH 8, a particle number density of 8.3 × 10^−3^ particles/nm^3^ was found, corresponding to about eight particles distributed in a cube with a volume of (10 × 10 × 10) nm^3^ (Figure 9).

One capped AuNP has a radius of 3.4 nm (2.4 nm core radius plus 1 nm capping radius [56]), which would show that neighboring AuNPs still have space without particle crowding. The determination of the interparticle distance represents an average which is only valid if AuNPs penetrate the whole brush. This is valid for the incubation of PMETAC in AuNP suspension at pH 8, but it cannot be applied on the PMETAC/AuNP composites at pH 4.

In fact, by comparing UV/Vis measurements of AuNP suspensions at different pH (Figure 5) with the measurements of PMETAC/AuNP composites (Figure 7), one can see that, except for the composite at pH 4, the absorption maxima appear at a higher wavelength for the AuNPs in brush than in suspension. The reason is a change in refractive index from water (*n* = 1.33) to polymer brush (*n* = 1.51) which leads to a red-shift. At pH 4, the change of refractive index can be neglected because the AuNPs are clustered in dispersion, and the plasmon coupling leads to a stronger red-shifted absorbance peak in the dispersion than in the brush.

**Figure 10:**
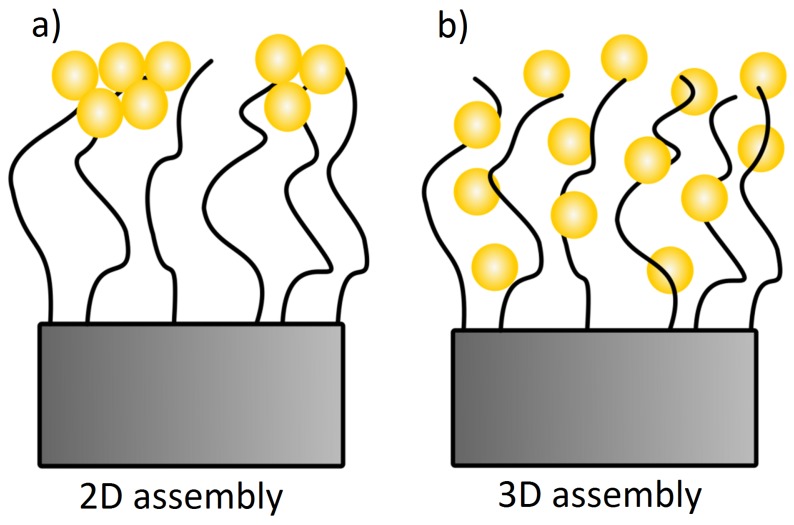
Two suggested assembly of brush/AuNP composites. Here, composites at pH 4 form more likely (**a**) a 2D assembly while composites at pH 8 form mainly (**b**) a 3D assembly.

By considering exclusively the composite materials, a higher particle uptake within the brush should lead to a red-shifting of the plasmon peak due to plasmon coupling, which is not the case (Figure 7). Here, a red-shift of the plasmon peak is observed for a lower particle uptake (at pH 4). This indicates a non-homogeneous distribution of AuNPs within the brushes after incubation at pH 4. A lower particle uptake and a simultaneous red-shift of the plasmon peak might indicate that the AuNPs are attached only on top of the brush surface (2D assembly). This would lead to a decreased interparticle distance, even though the averaged particle number density on the whole brush is lower. With respect to the results at pH 4, the higher particle uptake at pH 8 induces a plasmon peak at smaller wavelength. This indicates a more homogeneous distribution (3D assembly), which is related to deeper penetration in the brush. The interparticle distance is larger, due to the larger space available inside the brush than on the brush surface. Different assemblies at different pH can be therefore attributed to the AuNPs (Figure 4). AuNPs form clusters from suspensions at pH 4, which cannot penetrate into the dense polymer brush and get stuck on top of the surface, while single particles are observed at pH 8, which can penetrate the brush easily and are embedded (Figure 10). Furthermore, the attachment at pH 4 is stabilized by H-bonds due to the lack of charges, while the attachment at pH 8 is caused by the strong electrostatic attraction between deprotonated AuNPs.

In summary, sterical hindrance during incubation avoids a homogeneous distribution of AuNPs within the brush. In addition, increasing of the electrostatic interactions between AuNPs and polymer brush leads to a higher and deeper particle uptake.

## 5. Conclusions

The particle uptake of pH-dependent negatively charged AuNPs within strong positively charged PMETAC brushes was investigated at different pH. At low pH (pH 4), the AuNP form clusters in the dispersion used for incubation due to protonation of the acid groups in the AuNP coating and reduced electrostatic repulsion. The formation of clusters prevent the penetration of AuNP into the brush, which form clusters at the brush surface. The AuNP clusters are mainly bound to the brush via H-bonds. This assembly structure leads to a low amount of attached AuNPs, but to strong plasmon coupling between the AuNP clusters. At higher pH (pH 8), the acid groups are deprotonated, leading to highly charged AuNPs and a stabilized dispersion. The single AuNPs can penetrate into the brush and be physisorbed into the oppositely charged positive groups of the polyelectrolyte chains, leading to high uptake. In comparison to low pH, the plasmon coupling is reduced due to a more homogeneous distribution of the AuNPs.

To conclude, the study remarks that two effects are important for the uptake of AuNPs by polymer brushes: (1) the stability of the AuNP dispersion that is used for incubation and (2) the interaction between AuNPs and the polymer chains of the brush. The study shows how to control the AuNP distribution. Further work has to be done in order to tune a transition from 2D (AuNP layer) to 3D distribution (homomgeneous penetration) of the AuNPs. The results of the present study can be transferred to other assemblies of NPs (e.g., magnetic NPs) and polymer brushes.

## Figures and Tables

**Table 1:** Two-layer-model for fitting polymer brushes on a silicon wafer against air or water. Parameters are refractive index *n*, absorption coefficient *k* and the thickness of the layer, respectively. Empty arrays imply bulk properties with an infinity thickness and “fit”.

	Layer	*n*	*k*	Thickness (*nm*)
continuum	air/water	1/1.332	0	
1. layer	brush	fit	0	fit
2. layer	SiO_x_	1.500	0	1.5
continuum	Si	3.885	−0.020	

**Table 2:** Dry thickness of neat poly-[2-(Methacryloyloxy) ethyl] trimethylammonium chloride (PMETAC) brushes measured by ellipsometry at ambient conditions.

Sample	Thickness (nm)
1	28.04 ± 0.57
2	29.24 ± 0.32
3	27.11 ± 0.80
4	27.84 ± 0.77

**Table 3:** Comparison of ellipsometry and the atomic-force microscopy (AFM) full-indentation method for measuring the thickness in water for sample 1.

Method	Thickness
Ellipsometry	148.12 ± 6.42
AFM full-indentation	156.43 ± 9.44

**Table 4:** Measurements of the thickness in water of PMETAC/AuNP composites using the AFM full-indentation method. The thickness of the neat polymer brush was also listed for comparison reasons.

Neat PMETAC brush thickness (nm)	Composite at pH 4 thickness (nm)	Composite at pH 6 thickness (nm)	Composite at pH 8 thickness (nm)
156.43 ± 9.44	162.97 ± 6.03	170.68 ± 9.27	178.35 ± 11.77

**Table 5:** Particle number densities of AuNPs within PMETAC brushes obtained by UV/Vis of composites incubated at different pH.

Incubation medium	Absorbance before incubation	c before incubation (nmol/mL)	Absorbance after incubation	c after incubation (nmol/mL)	Δc (nmol/mL)	ΔN_total_ (Particles)	Particle number density (Particles/nm^3^)
at pH 4			0.574 ± 0.018	0.234	0.011	6.62 × 1013	0.0011
at pH 6	0.602 ± 0.006	0.245	0.455 ± 0.019	0.185	0.059	3.55 × 1014	0.0059
at pH 8			0.399 ± 0.021	0.162	0.083	4.98 × 1014	0.0083

## Supplementary Materials

Supplementary materials can be found at www.mdpi.com/2073-4360/8/4/134/s1.
