# Supplementary material for: Uptake of pH-Sensitive Gold Nanoparticles in Strong Polyelectrolyte Brushes
Source: Polymers (Basel). 2016 Apr 8;8(4):134. doi: 10.3390/polym8040134 (PMC6432499; doi:10.3390/polym8040134)
Supplement: Supplementary file 1 [file polymers-08-00134-s001.pdf]

Supplementary Materials: Uptake of pH-Sensitive Gold Nanoparticles in Strong Polyelectrolyte Brushes

Dikran Kesal, Stephanie Christau, Patrick Krause, Tim Möller and Regine von Klitzing*

Calculations of AuNP amount:

The mass of AuNP (m_{gold}) in 1 mL AuNP suspension is $(1.6 \pm 0.4)\times10^{-3}$ g, as derived by gravimetric analysis. In order to calculate the total amount of substance (n_{total}) of AuNPs in 1 mL suspension, the total number of particles (N_{total}) in 1 mL AuNP suspension has to be calculated. This was done by estimating the mass of a single AuNP. Assuming a spherical shape and an uniform face centered cubic (fcc) structure [47] with a AuNP radius (r) of 2.4 nm, the mass of a single AuNP m_{AuNP} was calculated:

$$V_{AuNP} = \frac{4}{3}\pi r^3 \tag{1}$$

$$m_{AuNP} = V_{AuNP} \cdot \rho_{fcc\ bulk} \tag{2}$$

where V_{AuNP} is the volume of one single AuNP with r = 2.4 nm and $\rho_{\text{fcc bulk}}$ is the density for fcc gold (19.3 g/cm^3). The mass is $m_{\text{AuNP}} = 1.18\times10^{-18}$ g. The toal number of particles N_{total} were calculated using:

$$N_{total} = \frac{m_{gold}}{m_{AuNP}} \tag{3}$$

Thus, the number of particles N_{total} in 1 mL AuNP stock suspension is 1.48×10^{15}. The amount of substance n_{total} in 1 mL AuNP stock suspension is 2.45 nmol and was calculated using the Avogadro constant $N_A = 6.022\times10^{23}$ mol^{-1}:

$$n_{total} = \frac{N_{total}}{N_A} \tag{4}$$

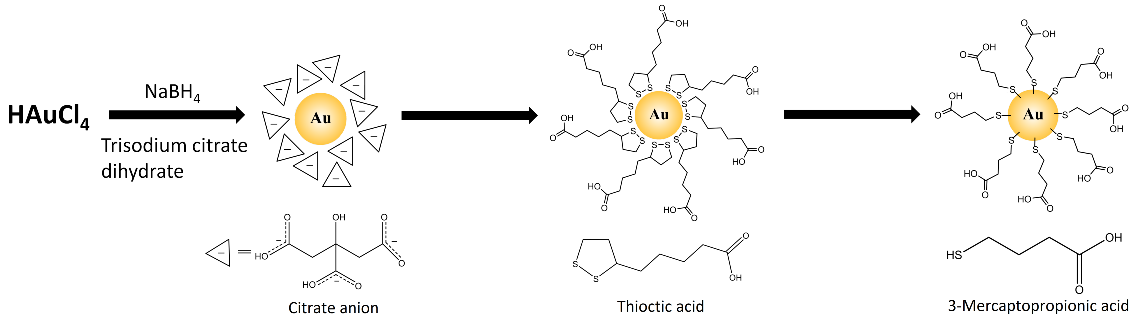

Figure S1. Schematic sketch of the three-step synthesis of AuNPs through ligand exchange reaction. As a first step $HAuCl_4$ is reduced by $NaBH_4$ in presents of trisodium citrate dihydrate to obtain citrate stabilized AuNPs. The citrate capping is exchanged by thioctic acid and afterwards by 3-mercaptopropionic acid to get the desired 3-MPA-capped AuNPs.

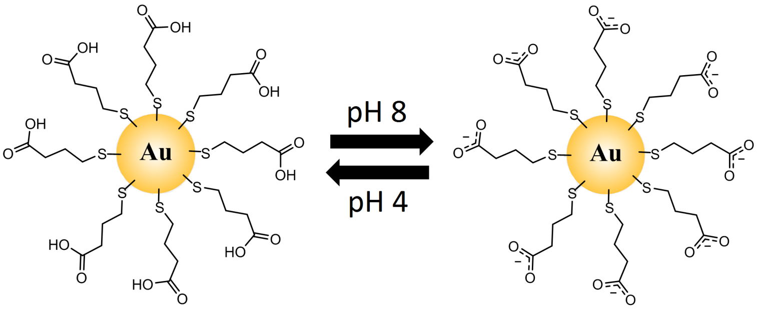

Figure S2. Varying the charge of the 3-MPA capping by changing the pH. pK_a of 3-MPA-capped AuNPs is 4.3. Exposing 3-MPA-capped AuNPs to pH 8 leads to a negatively charged surface due to the deprotonation of the carboxylic acid groups while exposing 3-MPA-capped AuNPs to pH 4 leads to uncharged AuNPs due to the protonation of the carboxylic acid groups.

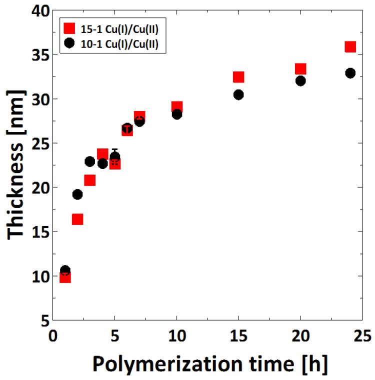

Figure S3. Tuning the PMETAC brush thickness by varying the $CuCl/CuCl_2$ ratio. The data are measured at ambient conditions using ellipsometry. The polymer thickness is increasing with polymerization time. Changing the Cu(I)/Cu(II) ratio gave no significant change.

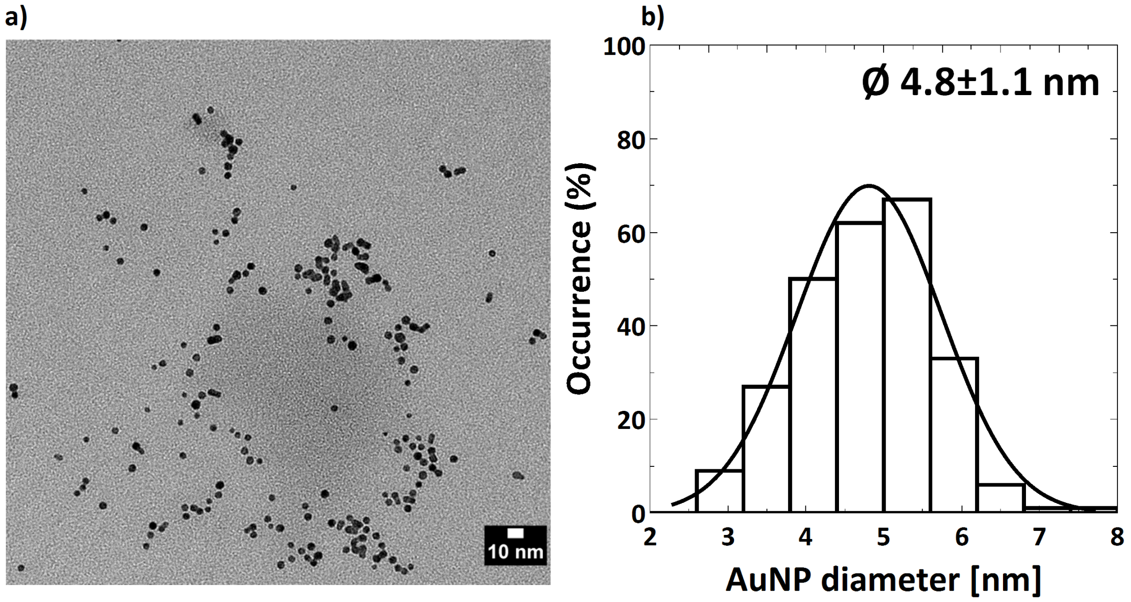

Figure S4. TEM image of 3-MPA-capped AuNPs (**a**) and the particle size distribution (**b**). The diameter of AuNPs are 4.8 ± 1.1 nm and was determined using ImageJ for the size determination.

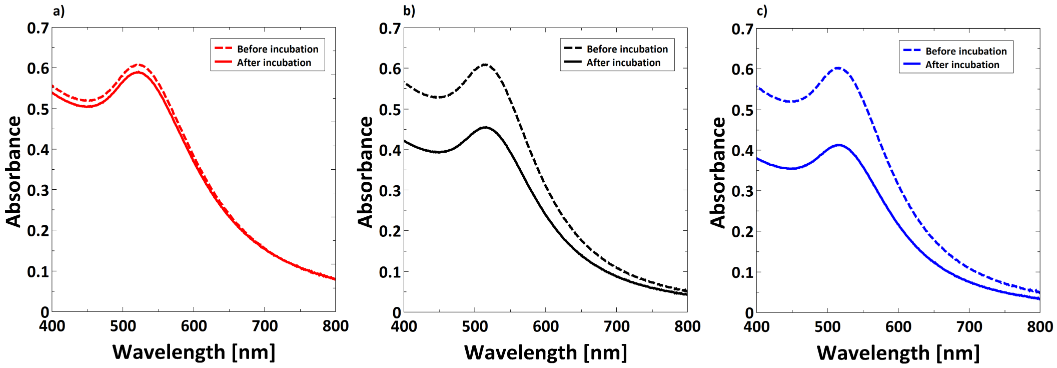

Figure S5. UV/Vis spectra before (dashed lines) and after (solid line) incubation of PMETAC brush in AuNP suspension. Shown for composites (**a**) at pH 4; (**b**) at pH 6; and (**c**) at pH 8. A decrease of the UV/Vis absorbance after the incubation was found which increases with increasing pH of the AuNP incubation suspension. The difference before and after the incubation is equal to the amount of AuNPs within the brush since the UV/Vis absorbance is proportional to the concentration of the AuNP suspension.
